# The role of dendritic spines in epileptogenesis

**DOI:** 10.3389/fncel.2023.1173694

**Published:** 2023-08-02

**Authors:** Gary Jean, Joseph Carton, Kaleem Haq, Alberto E. Musto

**Affiliations:** ^1^Medical Program, School of Medicine, Eastern Virginia Medical School, Norfolk, VA, United States; ^2^Department of Pathology and Anatomy, Eastern Virginia Medical School, Norfolk, VA, United States; ^3^Department of Neurology, Eastern Virginia Medical School, Norfolk, VA, United States

**Keywords:** epilepsy, dendritic spine, epileptogenesis, neuroinflammation, neural plasticity

## Abstract

Epilepsy is a chronic central nervous system (CNS) disease associated with high morbidity. To date, there is no known disease-modifying therapy for epilepsy. A leading hypothesis for a mechanism of epileptogenesis is the generation of aberrant neuronal networks. Although the underlying biological mechanism is not clear, scientific evidence indicates that it is associated with a hyperexcitable synchronous neuronal network and active dendritic spine plasticity. Changes in dendritic spine morphology are related to altered expression of synaptic cytoskeletal proteins, inflammatory molecules, neurotrophic factors, and extracellular matrix signaling. However, it remains to be determined if these aberrant dendritic spine formations lead to neuronal hyperexcitability and abnormal synaptic connections or whether they constitute an underlying mechanism of seizure susceptibility. Focusing on dendritic spine machinery as a potential target for medications could limit or reverse the development of epilepsy.

## 1. Introduction

Epilepsy is a chronic central nervous system (CNS) disease characterized by a state of spontaneous unprovoked recurrent seizures due to aberrant synchronous electrical activity (Fisher et al., 2014). It is associated with high morbidity, and certain types of epilepsy such as temporal lobe epilepsy (TLE) and post-traumatic epilepsy can be notoriously difficult to control pharmacologically. To date, the vast majority of pharmacological interventions aim to control the symptoms of epilepsy by modulating neuronal activation (Hakami, 2021). Epileptogenesis is the process by which a brain insult or other unknown factors initiate a cascade of pathophysiological changes (e.g., neural loss, chronic neuroinflammation, reactive gliosis, altered gene expression, etc.) that results in a chronic epileptic state (Pitkänen et al., 2015; Webster et al., 2017). Understanding this process will enable the discovery of novel therapeutic targets that may block an underlying cause of epileptogenesis and therefore prevent epilepsy. This review focuses on new research updates in neuroplastic changes in the epileptic brain, specifically elucidating how different aspects of dendritic spine plasticity could contribute to epileptogenesis.

## 2. Dendritic spines and epilepsy

It has long been hypothesized that an alteration in the normal excitatory and inhibitory ratio plays a role in the development of epilepsy. Dendritic spines are mostly observed in excitatory synapses providing a strong influence on neural network activity (Ofer et al., 2022). Multiple studies in both human TLE patients and animal models of epilepsy have demonstrated hippocampal pyramidal neuron dendritic spine loss, synaptic remodeling, and aberrant plasticity (Raijmakers et al., 2016; Yuan et al., 2016; Blazejczyk et al., 2017; Urban et al., 2020). Dendritic spine loss and swelling is seen within minutes after seizures followed by an apparent recovery to levels similar to control. However, in models of chronic epilepsy, there is evidence of irreversible damage resulting in changes in dendritic spine morphology and density (Guo et al., 2012; Musto et al., 2015; Musto et al., 2016). These findings suggest that the alteration of dendritic spines is positively correlated with epileptogenesis, however, it remains a challenge to determine whether dendritic spine abnormalities are a cause of epilepsy or simply a consequence of recurrent seizures (Wong and Guo, 2013). This difficulty has prompted many different approaches to identify how different variables influence dendritic spine plasticity.

**FIGURE 1:**
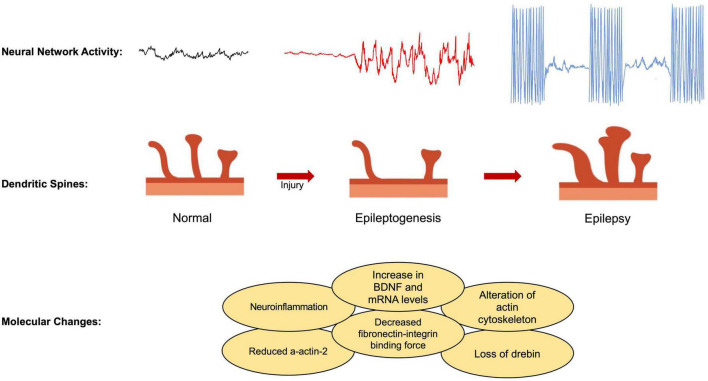
Illustrations of neural network activity and dendritic spine morphology with progression from normal, epileptogenesis, and epilepsy states. Molecular changes seen in epileptogenesis and epilepsy.

Here, we review different aspects of the dendritic spine modification that could contribute to epileptogenesis (Figure 1).

### 2.1. Structural changes

Precisely how dendritic spines undergo changes after seizures that may contribute to chronic epilepsy remains a topic under intense study. Dendritic spine loss occurs after seizures, but how spine loss contributes to seizure generation remains a mystery. Numerous studies have attempted to explain this process. A 2007 landmark study evaluated acute changes *in vivo* using kainate injected mice, finding that dendritic beading and swelling occurred rapidly within the first hour following tonic-clonic seizures, with obliteration of approximately 50% of dendritic spines. Partial recovery occurs over the following few hours with residual injury persistent up to 24 h (Zeng et al., 2007).

Rossini et al. (2021) studied dendritic morphology from 28 human samples with various drug resistant epilepsy types, including Focal Cortical Dysplasia (FCD) Types 1a, 2a, 2b, temporal lobe epilepsy with hippocampal sclerosis (TLE-HS), and cryptogenic epilepsy, and compared them to autopsy specimen without history of seizures. Of note, within the lesional core of Type 2 FCD, there was a heavily disorganized network of thick dendrites originating from dysmorphic neurons, interspaced with small pyramidal neurons showing simplified dendritic arborization. Dendritic area, branch points, and complexity did not show any significant difference except for a reduction in dendritic branching in TLE-HS. Similar dendritic morphology and density were noted in control, cryptogenic, TLE-HS, Type 1a FCD, and Type 2 FCD perilesion. In contrast, within the core of Type 2 FCD there was dramatically reduced spine density. Furthermore, perisomatic baskets were evident with filopodia-like protrusions (Rossini et al., 2021). These observations demonstrate significant rearrangement in neuronal morphology and synaptic network in epilepsy, however, since perilesional cortex was largely normal appearing, chronic seizures themselves may not necessarily cause dendritic pathology. Some limitations to this conclusion include the advanced age of controls as it is known that dendritic loss is a normal consequence of aging.

Investigating the changes to inhibitory GABAergic activity may help expand our understanding of the biphasic neuronal response to seizures. One study examining pilocarpine induced status epilepticus in rats found initial loss of GABAergic synapses in the dentate gyrus granule cell and molecular layer, with subsequent rebound above control levels. The most significant changes were noted in the outer molecular layer, with loss of axon-spine synapses and subsequent proliferation of axon-spine and axon-shaft synapses. Looking at GABA negative synapses, which were presumed to be mostly glutamatergic, similar initial loss was seen followed by restoration to near control levels. Interestingly, however, there were up to twice the number of perforated synapses in epileptic rats, which are known to contain a high number of glutamate receptors (Thind et al., 2010). Instead of looking directly at the synapse itself, a separate study found a correlation between loss of GABAergic interneurons within the dentate gyrus granule cell layer and seizure frequency in pilocarpine treated mice (Buckmaster et al., 2017). Although a definitive explanation for these seemingly contradictory findings has yet to be found, some have theorized that the abundant inhibitory synapses are dysfunctional, as evidenced by dysfunctional chloride channels and GABA receptors in inhibitory neurons in models of epilepsy (Mele et al., 2019). Altered excitatory-inhibitory ratio in epilepsy remains a prime culprit in epileptogenesis.

Dentate granule cells and its projections, the Mossy Fibers, are critical in the learning and memory process and are implicated in epilepsy. Granule cells in epilepsy models are known to undergo significant change. These changes include mossy fiber sprouting, migration to ectopic locations, and formation of new, disorganized dendrites (Hester and Danzer, 2014; Leifeld et al., 2022). These mossy fiber changes are related to dendritic spine morphological changes, as development of new widely spread dendrites and creation of new dendritic spines with increased density in areas proximal to mossy fiber sprouting are observed during migration of developing granule cells (Puhahn-Schmeiser et al., 2022). Interestingly, initial dendritic spine loss after seizures is transient, followed by a spine regeneration or recovery, suggesting that the dendritic spine changes observed in chronic epilepsy reflect ongoing plasticity (Beamer et al., 2018). On the other hand, immature granule cells appear to form large gyri and are more susceptible to aberrant plasticity (Magagna-Poveda et al., 2017). However, those immature granule cells are also better able to integrate appropriately following seizure insult (Gao et al., 2015; Althaus et al., 2019). This evidence could suggest that projections of immature neurons in the dentate gyrus are susceptible to restore the neural circuitry as a consequence of neural damage, but it remains to be explored if such connectivity is properly functional (Table 1).

**TABLE 1:** Summary of changes in dendritic spine morphology or density in experimental epilepsy.

Authors	Model	Dendritic spine changes
Beamer et al., 2018	Kainate, 10-week-old C57BL/6 mice	Increased spine density and volume, increased mushroom/non-mushroom spines in anti-microRNA-22 inhibition + SE group.
Blazejczyk et al., 2017	Kainate, 3-month-old Wistar rates	Increased filopodia and decreased spine density in Elmo1 overexpression group.
Gao et al., 2015	Pilocarpine, 6–7-week old male Sprague-Dawley rats	Increased mushroom-like spine density in outer molecular layer of granule cells formed after SE.
Guo et al., 2012	Kainate, 2–3-month old C57BL/6 transgenic mice	Immediate spine loss and dendritic beading, amount of spine loss dependent on duration of seizure. Mild partial recovery over 4 h period.
Guo et al., 2016	Kainate, 2–3-month old C57BL/6 transgenic mice	Acute dendritic beading and spine loss followed by mild recovery over 6 weeks, with 50% loss at 6-weeks. mTOR inhibitor pretreatment showed significantly less spine loss and less dendritic beading.
He et al., 2010	Pilocarpine, male and female mice B6	Spine loss in third and fourth order interneuron dendrites in dorsal subiculum. Increased spine density in second and third order pyramidal dendrites in dorsal subiculum.
Kurz et al., 2008	Pilocarpine, adult Sprague-Dawley rats	Spine loss, decreased spine density in dentate gyrus. Mature spines not affected.
Musto et al., 2015	Pilocarpine, C57BL/6 adult male mice	Dendritic swelling and beading, decreased spine density in CA1 and DG at 7 days post SE.
Musto et al., 2016	Pilocarpine, C57BL/6 adult male mice	Increased filopodia spines. Decreased spine density and spine length.
Peng et al., 2021	Lithium-pilocarpine, Sprague-Dawley rats	Dendritic spine loss, decreased density in CA1, CA3, dentate gyrus.
Puhahn-Schmeiser et al., 2022	Kainate, C57BL/6, Reeler, p35 KO	Increased spine density in granule cell layer, particularly basal dendrites. Longer dendrites with increased branching.
Smilovic et al., 2022	C57BL/6J, TNF KO	Reduced spine density, larger spines, spine heads in TNF KO.
Tiwari et al., 2020	Kainate, C57BL/6J, Kv4.2 KO mice	Decreased spine density, decreased thin and stubby type.
Yuan et al., 2016	Lithium-pilocarpine, C57BL/6 mice	Predominance of thin and filopodia type spines.
Zeng et al., 2007	Kainate kindling, C57BL/6 mice	Dendritic spine beading, generalized loss early, partial recovery at 24 h.

### 2.2. Molecular machinery

It has long been known that the mechanistic target of rapamycin, mTOR, is heavily involved in dendritic spine plasticity via dual Ras-PI3k-mTOR and Ras-MAPK pathways (Kumar et al., 2005). Much effort has since been made to further understand the influence of mTOR on epileptogenesis and related processes Inhibiting mTOR through rapamycin pretreatment rescued the reduced distal dendritic branching and spine density observed following pilocarpine induced status epilepticus, though the effect in higher order branches was unable to be determined due to extensive loss (Brewster et al., 2013). Another study showed that rapamycin pre-treatment of mice undergoing kainate-induced seizures significantly reduced the dendritic spine beading and loss seen without treatment. Post-seizure treatment with rapamycin, however, only demonstrated significant improvement in the chronic phase 4–6 weeks after induction. The authors noted that rapamycin reduced actin depolymerization and phosphorylation of cofilin, proteins important in regulating dendritic structure and thus contributing to persistent neural networks (Guo et al., 2016).

Long-term potentiation is described as the formation of a persistent neural network associated with learning and memory formation. While this is an inherently physiological process in healthy individuals, aberrant long-term potentiation (LTP)-like synaptic plasticity is thought to be a key phenomenon seen in many epilepsy syndromes. One study noted that mTORC1 signaling is necessary for long-lasting strengthening of the excitatory synapse. Dendritic changes were most notable on mature mushroom type spine morphology, where activation of mTORC1 in conjunction with sub-threshold synaptic stimulation resulted in significant enlargement of spine heads, a sign of enhanced excitatory synapse formation (Henry et al., 2017). As a result, the mTORC1 pathway may be an interesting target for future pharmacologic interventions aimed at preventing epileptogenesis.

Multiple studies have sought to determine the difference in proteins expressed in hippocampal regions undergoing epileptogenesis, specifically those playing a role in synaptic and dendritic spine plasticity. A recent study investigating protein expression in epileptic hippocampi found 27 differentially expressed proteins with a fold change >1.5 when compared to control, many of which are involved in regulating synaptic plasticity and calmodulin-dependent kinase activity. Two proteins were significantly upregulated: CaMKII-a, essential for synaptic plasticity, and CaMKII-b, which has F-actin binding properties (Qian et al., 2022). α-Actinin-2 has been shown to be reduced in the inner molecular layer 1–2 weeks post-status epilepticus at the stage of granule cell spinogenesis and morphogenesis and persisted at the chronic stage when new functional synapses were forming (Sbai et al., 2021). Previous studies have shown that this protein is necessary for formation of actin bundles in hippocampal dendritic spines (Hodges et al., 2014), suggesting that the absence of α-actinin-2 affects the organization of actin filaments leading to plasticity to dendritic spine density and shape.

Following dendritic spine injury, recovery is at least partially dependent on actin depolymerization, mediated by cofilin dephosphorylation, itself a calcineurin mediated process. Administration of calcineurin inhibitors, tacrolimus and cyclosporine A, significantly reduced status epilepticus induced dephosphorylation of cofilin, cofilin-actin binding, and, ultimately, granule and pyramidal cell dendritic spine loss (Kurz et al., 2008).

Actin is a critical protein for generating structural support for dendrites and is regulated by actin binding proteins, of which drebin is a key player. In Drebin-KO mice, inducing epileptiform activity through suppression of Mg^2+^ in the medial entorhinal cortex significantly increased seizure susceptibility, indicating that loss of drebin may lead to altered synaptic connectivity and excitatory-inhibitory balance via reduced actin cytoskeleton stability (Klemz et al., 2021). Actinfilin, another actin binding protein specific to F-actin in neurons which is closely linked to infantile spasms when deficient or absent, was noted to regulate dendritic spine enlargement. Actinfilin-KO mice showed narrowed dendritic head width and reduced synaptic formation and a 50% reduction in F-actin (Hu et al., 2020).

### 2.3. Neuroinflammatory-immunity

Growing evidence points toward neuroinflammatory mechanisms underlying epileptogenesis (Rana and Musto, 2018). Microglia in particular are thought to play a central role as they are rapidly activated following acute convulsive stimuli, releasing proinflammatory cytokines which are thought to contribute to a hyperexcitable state (Hiragi et al., 2018). Microglia, however, are also known to provide pro- and anti-epileptogenic effects based on individual phenotype and seizure mechanism (Eyo et al., 2017; Kinoshita and Koyama, 2021).

One study found in kainate injected mice, there was extensive dendritic spine loss in dentate granule cells as well as in CA1 and CA3 pyramidal cells in the acute and early chronic stages of status epilepticus (Xie et al., 2021). This was accompanied by an increase in M1 microglia acutely, followed by a transition to M2 microglia. This is consistent with the dual role of microglia in acute and chronic inflammation, as microglia-depleted mice demonstrated worsened seizure severity as little as 20 min after induction and experienced increased seizure number and duration through a 31-day observation period (Wu et al., 2020). Further evidence is provided by observing microglia-dendrite interactions *in vivo* using photon microscopy. Microglia preferentially interfaced with dendritic beads forming what was termed microglial process pouches, similar in morphology to phagocytic engulfing but persisting well beyond the timeline of phagocytosis with the majority of pouches stable at 4 h without demonstrating lysosomal activity (Eyo et al., 2021). Additionally, microglia-dendritic bead interaction was shown to resolve more quickly than dendritic beads alone, suggesting that dendritic remodeling by microglia is a possible neuroprotective mechanism.

Complement proteins are immune components closely related with microglia and its role in epileptogenesis has become a target for further study. In general, a key role of complement proteins is opsonization to aid in destruction by other immune cells, particularly microglia. Notably, C1q, the initiating protein in the classical complement pathway, is known to attract microglia to excess dendritic spines in a process known as dendritic pruning. In C1q KO mice dendritic branching, length and spine density were enhanced, particularly in thin spines. Since no seizures were induced it is difficult to draw conclusions about the pro- vs. anti-epileptic effects of these findings, but it is clear that the complement pathway has a role in regulating dendritic plasticity (Ma et al., 2013).

Further investigation into the relationship between dendritic spine loss and complement-microglia activation points to aberrant microglial activity. In a human cohort of patients with medically refractory epilepsy (cryptogenic, TLE-HS, and focal cortical dysplasia types IIa and IIb), FCD type IIb samples were significantly associated with dendritic spine loss, C1q, C3 activation, and microglial activity. Double labeling confirmed direct association between complement activation and microglial activity. Furthermore, microglia were primarily found in their inactive state around the soma of pyramidal neurons in settings of normal spine density, whereas they were mostly in their active state and frequently in contact with dendrites in settings of spine loss (Rossini et al., 2022). A similar earlier study found that complement mediated microglia activation increased in a sample of human TLE-HS as well (Wyatt et al., 2017). Despite the discordant results, these studies suggest pathologic reactivation of microglia as a potential factor in epileptogenesis.

Tumor necrosis factor (TNF)-alpha is a proinflammatory cytokine shown to influence homeostatic synaptic plasticity (Heir and Stellwagen, 2020). Dentate granule cells with constitutively decreased TNF-α display decreased dendritic spine density and increased spine size, changes similar to entorhinal denervation (Smilovic et al., 2022). However, perhaps due to its dual effects in inflammation-mediated damage and repair, blanket inhibition of TNF-α or similar proinflammatory mediators such as IL-1 or IL-6 have promising yet inconsistent antiepileptogenic effects, especially in humans (Klein et al., 2018; Costagliola et al., 2022).

### 2.4. Neurotrophic factors

Several neurotrophic factors contribute to epileptogenesis. Of these, Brain Derived Neurotrophic Factor (BDNF) is the most relevant to the development and maintenance of dendritic spines and related synaptic structures following an epileptogenic event. BDNF is associated with mossy fiber sprouting in TLE and its levels are elevated in animal models of TLE. In a recent study, both BDNF and mRNA levels were shown to increase in CA2 and dentate gyrus and were associated with granule cell layer dispersion, mossy fiber sprouting, and epileptiform activity. During kainate induced seizures, BDNF was shifted to a transcriptionally active form located in the center of the nucleus (Tulke et al., 2019). The same location shift was observed in a model of LTP with chemically activated neurons (Skupien-Jaroszek et al., 2021). Together, these findings suggest that BDNF’s contribution to synaptic plasticity and neuronal activation through aberrant connections in the hippocampus could be a target for dendritic plasticity in epileptogenesis.

Evidence has shown that blocking BDNF is associated with decreased dendritic spine density, increased spine length, and decreased dendritic head width (Kellner et al., 2014). This study examines BDNF’s effects on hippocampal dendritic spine morphology at various developmental stages. The results demonstrate that BDNF is necessary for *in vitro* activity-dependent preservation of the mature spine phenotype and plays a role in promoting neurite development. Moreover, another study explains the role of BDNF in regulating dendritic spine number, structure, and plasticity (Zagrebelsky et al., 2020). LTP and long-term depression (LTD) are linked to structural alterations of dendritic spines, such as spine head enlargement or shrinkage. For learning and memory formation, spine structural plasticity is necessary and is dependent on intracellular signaling cascades. LTP must be induced and maintained in order for learning and memory to occur, and BDNF is essential for both of these processes. BDNF signaling is also implicated in cytoskeletal remodeling, a crucial component of spine plasticity.

Changes to BDNF have also shown to cause pruning and enlargement of dendritic spines (An et al., 2008). Proteases convert dendritically translated pro-BDNF into mature BDNF, which then activates TrkB in an autocrine manner on activated spines. Stimulation then causes local synthesis of proteins involved in spine growth and plasticity, which promotes the expansion of the spine head and the development of stable LTP. Parallel to this, adjacent diffused pro-BDNF promotes pruning of unstimulated spines by strongly attaching to the sortilin/p75NTR receptor complex. The study also explains the suppression of dendritic local BDNF production impacts early-phase LTP but not basal transmission in apical dendrites. This selective impairment in dendritic LTP is consistent with the size reduction in spine heads observed on apical dendrites of CA1 neurons in Bdnfklox/klox mice.

### 2.5. Extracellular matrix

The extracellular matrix is a network of structural scaffolding that provides support to neurons and facilitates intercellular communication. As such, it stands to reason that changes in ECM structure and composition may contribute to epileptogenesis. Integrins are a family of proteins that connect cells to the extracellular matrix and play a key role in cell signaling and extracellular-intracellular communication (Danen, 2000-2013), contributing to neuroplasticity. It is well known that integrins are critical for regulation of excitatory neuroplasticity, however, newer evidence also shows an important role in inhibitory GABAergic neuroplasticity as well (Wiera et al., 2022), indicating that integrins can alter the excitatory-inhibitory ratio that can influence the generation of an epileptic state.

Fibronectins are extracellular proteins that bind cell surface integrin receptors and induce various downstream effects including transcription factor activity, gene induction, and protein modification (Dalton and Lemmon, 2021). An experiment studying fibronectin-integrin receptor signaling found decreased fibronectin-integrin binding force in epileptic mice dentate gyrus granule cells. Pretreatment with a5b1 integrin antibodies partially restored membrane integrin receptor function. Patch clamp recordings showed that fibronectin inhibited GABA currents while Arginine-Glycine-Aspartate (RGD) peptides, which block fibronectin-integrin binding, enhanced GABA current. Treatment with a5b1 integrin antibody was also noted to reduce epileptiform discharges induced by aminopyridine (Wu et al., 2017). Conversely, the Wiera study referenced above found that inhibiting B1 vs. B3 integrin interfered with long-term NMDA dependent GABAergic plasticity in CA1 neurons. The role of integrins in neuronal plasticity is just starting to be elucidated but may serve as a novel therapeutic target in resistant epilepsy.

### 2.6. Electrical currents

Changes in electrical activity within the hippocampal neuronal network is thought to be the fundamental change that leads to TLE. In an effort to elucidate the precise mechanism behind this process, one study examined electrophysiologic activities associated with morphologic changes in the subiculum-hippocampus network using mouse pilocarpine model of status epilepticus (He et al., 2010). As with previous studies, significant neuron loss was noted alongside increased dendritic spine density in pyramidal neurons. This correlated with increased up-down activity in the 0.3–1.0 Hz range and increased amplitude gamma activity in dorsal subiculum neurons. Microepileptiform activity was correlated with dendritic spine loss and aberrant dendritic spines (Musto et al., 2015). A potential explanation to this finding is that dendritic spines are elementary voltage compartments with the ability to activate independently from the rest of the neuron (Cornejo et al., 2021). In the context of epileptogenesis, where numerous aberrant synapses are forming, it is possible that the change in dendritic spine morphology and number could contribute to abnormal electrical activity. However, future studies should focus on whether this abnormal electrical activity is a cause or consequence of aberrant dendritic spines.

Inhibitory factors are the other side of the excitation-inhibition ratio that is thought to underlie the generation of seizure activity. While much of the research focus has been on increased glutamatergic burst activity as the initial seizure generator, somewhat paradoxically, inhibitory GABAergic activity has also been implicated in ictogenesis, consistent with observed low voltage fast activity seen at the onset of certain subtypes of seizures in human and animal models (Avoli et al., 2016; de Curtis and Avoli, 2016).

GABAergic activity has been shown to be a primary driver in synaptic plasticity through dendritic spine selection. In rat hippocampal CA1 cultured slices, a study in 2013 found that dendritic spine shrinkage and elimination is mediated by local GABAA input through suppressing intracellular [Ca^2+^] in a preictal spike-timing dependent manner (Hayama et al., 2013). Another study in female adolescent mice showed that inhibitory GABAA receptors are responsible for triggering synaptic pruning to eliminate excess synapses. In mice that were genetically modified to lack this response, synaptic pruning did not occur and the mice were unable to learn to avoid unwanted stimuli when the location changed (Afroz et al., 2016). Inhibitory processes are closely involved in the regulation of the formation of long-term electrical networks through their ability to modify dendritic connections.

Interneurons are primarily inhibitory regulators of principal cells in the hippocampus that are thought to contribute to the hyperexcitability characteristic of seizures. Thus, loss of these interneurons is thought to contribute to unopposed excitability leading to seizure activity, but this tempting idea has been contradicted by numerous studies. In a study of human lesional resistant epilepsy samples, despite the expected loss in neuron number, overall inhibitory perisomatic input to neocortical pyramidal layers 2/3 was preserved in epileptic tissue when compared to matched non-epileptic samples (Tóth et al., 2021). They did note, however, that parvalbumin stained interneuron perisomatic boutons were enlarged, enabling greater neurotransmitter release and therefore greater synaptic transmission, possibly contributing to increased synchrony. An earlier study found that in comparison to physiologic synchronous activity, interneurons had greater contribution than principal cells in epileptiform activity, particularly in the initiation phase (Kandrács et al., 2019).

The interplay between excitatory and inhibitory neurons may also contribute to network reorganization and LTP. In mature rat CA1 neurons, induction of LTP through theta burst stimulation leads to loss of both excitatory and inhibitory synapses and a compensatory enlargement of remaining synapses (Bourne and Harris, 2011). Another study using low frequency stimulation combined with MNI-glutamate uncaging on single excitatory dendritic spines in cultured hippocampal neurons led to weakening of local GABAergic synapses with compensatory strengthening in more distant inhibitory synapses. These findings suggest repeated stimulation may lead to formation of aberrant networks that predispose to synchronous electrical activity. For example, interneuron-interneuron inhibition may lead to firing of excitatory principal neurons along a preformed pathway that results in dysregulated neuronal firing (Ravasenga et al., 2022).

Another way interneurons and inhibitory activity may lead to generation of spontaneous seizures is through generation of pathologic high frequency oscillations and maintenance of interictal discharges. The association between pathologic fast ripple/high frequency oscillations and seizures has long been established (Jiruska et al., 2017). Inhibitory interneuron interaction with pyramidal neurons through feedback and reciprocal inhibition is known to play a role in rhythm generation and synchronizing ripple activity. Furthermore, interneurons are necessary to pace excited pyramidal neurons to continually generate fast ripple activity (Stark et al., 2014).

Ion channels are critical modulators of electrical activity and synaptic transmission. Kv4.2, a voltage gated potassium channel, has been implicated in epilepsy and seizure onset (Gross et al., 2016; Hong et al., 2018; Bavan et al., 2022). In a mouse model with reduced expression of Kv4.2, there was notable decreased dendritic spine density in CA1 despite increased seizure susceptibility using models of epilepsy. Moreover, there was an increase of theta power and epileptiform spikes. Restoring Kv4.2 to normal physiologic levels eliminated this difference, and overexpression had a corresponding increase in seizure latency, indicating the role of Kv4.2 in regulating neuronal network excitability. Of note, dendritic morphology favored filopodia and thin type rather than mushroom type (Tiwari et al., 2020).

## 3. Conclusion and future directions

Current research has shown that following neuronal injury and/or changes in synaptic plasticity, significant changes occur to morphology of dendritic spines and the surrounding synapse which can contribute to epileptogenesis. During this phase, multiple aspects involved in spine architecture can be abnormally expressed in neural tissue, including proteins, microglia, neurotrophic factors, and integrins. This altered excitation/inhibitory balance contributes to a state of synchronous hyperexcitability and elicits abnormal electrical activity leading to development of additional seizures which creates vicious cycle of damage and further alterations to dendritic spine morphology. Despite this, however, it remains difficult to determine conclusively whether it is the changes in dendritic spines themselves that contribute to the epileptic state or if they are merely a consequence of an underlying process. New experimental methodology needs to be developed to answer this question. Currently, the only pharmacologic interventions for epilepsy modulate neuronal activation to block symptomatic seizures, however by gaining an understanding of the underlying cause of dendritic spine morphological change a target can be found for novel treatment options which can potentially reverse epilepsy during the process of epileptogenesis.

The mTOR pathway is one such target that has shown some promise. Despite extensive study, however, mTOR inhibitors, such as rapamycin, sirolimus, and everolimus have demonstrated limited clinical benefit due to inconsistent results and significant side effects due to multiple downstream effects (Karalis and Bateup, 2021). Many other compounds targeting various mechanistic targets remain in the preclinical phase (Ots et al., 2023). For example, the soluble epoxide hydrolase receptor, through inhibition by TPPU pretreatment, was found to rescue dendritic spine loss and attenuate seizure severity (Peng et al., 2021). Targeting neuroprotection or inflammatory mechanisms could also be a mechanistic target of dendritic spine damage in epilepsy (Musto et al., 2015, 2016; Pototskiy et al., 2021). The variety of targets studied is an indication that understanding of and identifying a common mechanism of epileptogenesis is still under development. A common theme is that maximal antiepileptogenic effect is achieved by pretreatment before induction of seizures, suggesting that preventing early modification of dendritic spines can block hyperexcitability and network reorganization and, consequently, the seizure susceptibility.

## Author contributions

AM conceived the idea, concept, and wrote and reviewed the manuscript. GJ, JC, and KH wrote and reviewed the manuscript. All authors contributed to the article and approved the submitted version.
